# Supplementary material for: Monitoring the influx of new species through citizen science: the first introduced ant in Denmark
Source: PeerJ. 2020 Apr 8;8:e8850. doi: 10.7717/peerj.8850 (PMC7150537; doi:10.7717/peerj.8850)
Supplement: Supplemental Information 1 [file peerj-08-8850-s001.doc]

|  | ***T. caespitum*** | |  | ***T. immigrans*** | |  | **Wilcoxon test** | | |
| --- | --- | --- | --- | --- | --- | --- | --- | --- | --- |
|  | **Mean** | **SD** |  | **Mean** | **SD** |  | **W** | **p** |  |
| Annual mean temperature | 8.24 | 2.3 |  | 11.39 | 2.26 |  | 16085 | < 0.001 | *** |
| Temperature seasonality | 6725.85 | 716.6 |  | 6819.02 | 800.18 |  | 50062 | 0.002 | ** |
| Mean temp. of warmest quarter | 16.75 | 2.49 |  | 20.04 | 1.96 |  | 14780 | < 0.001 | *** |
| Mean temp. of coldest quarter | -0.48 | 2.59 |  | 2.55 | 3.16 |  | 24930 | < 0.001 | *** |
| Annual precipitation | 847.17 | 233.88 |  | 727.33 | 214.78 |  | 79919 | < 0.001 | *** |
| Precip. seasonality | 26.47 | 9.74 |  | 27.39 | 10.92 |  | 57526 | 0.5829 |  |
| Precip. of wettest quarter | 285.29 | 70.82 |  | 246.85 | 74.7 |  | 79256 | < 0.001 | *** |
| Precip. of driest quarter | 152.18 | 56.53 |  | 126.77 | 49.74 |  | 75176 | < 0.001 | *** |
| Precip. of warmest quarter | 261.61 | 81.23 |  | 186.07 | 67.11 |  | 89967 | < 0.001 | *** |
| Precip. of coldest quarter | 171 | 63.51 |  | 171.13 | 75.58 |  | 61535 | 0.4136 |  |
