# Supplementary figures and images for: Monitoring the influx of new species through citizen science: the first introduced ant in Denmark
Source: PeerJ. 2020 Apr 8;8:e8850. doi: 10.7717/peerj.8850 (PMC7150537; doi:10.7717/peerj.8850)

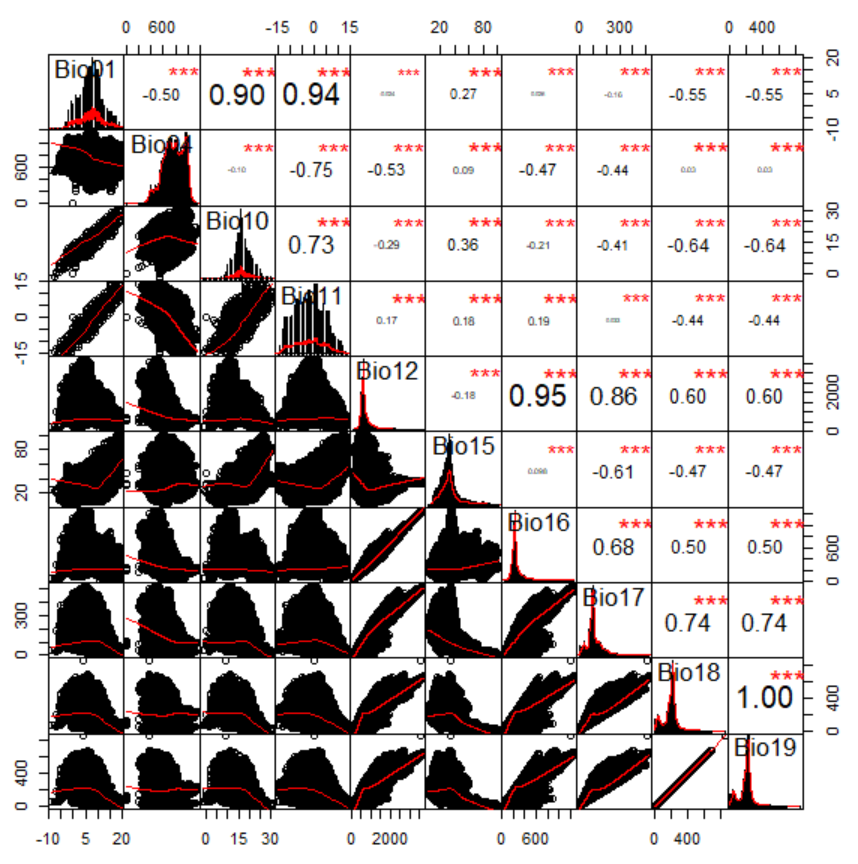

Supplement: Supplemental Information 2 [file peerj-08-8850-s002.png]

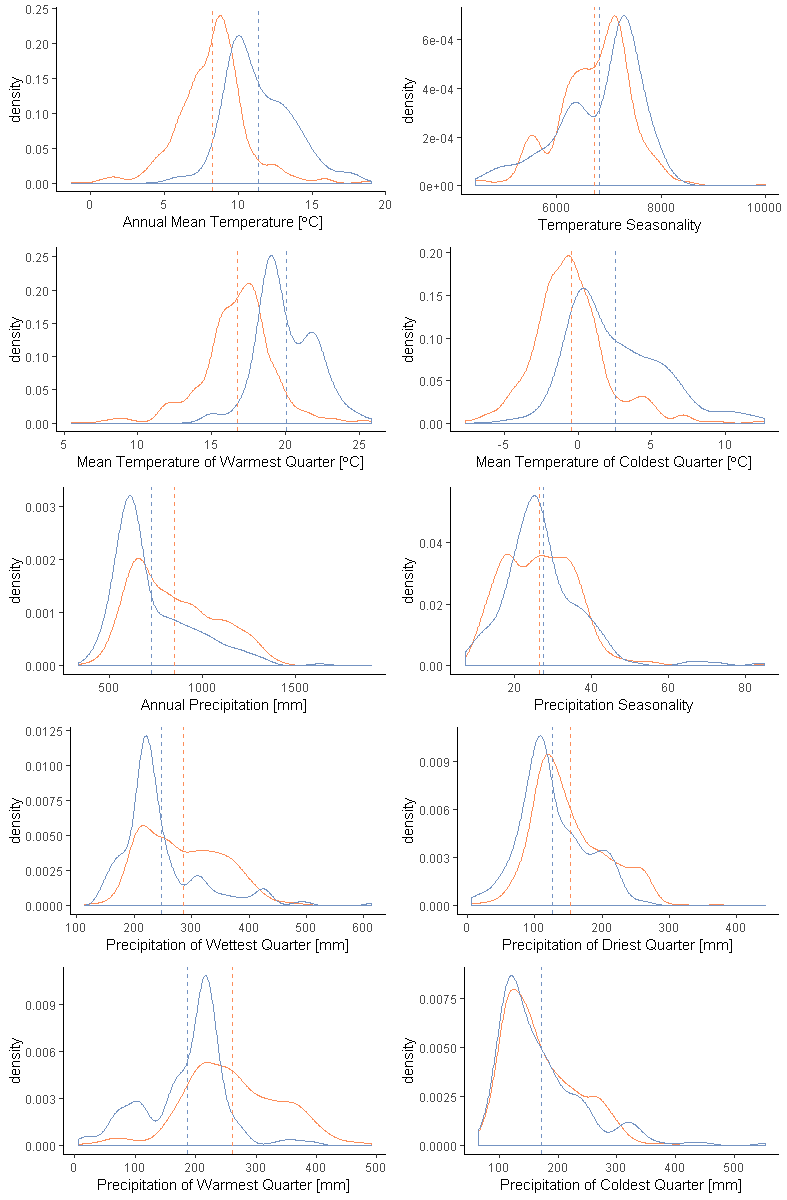

Supplement: Supplemental Information 3 [file peerj-08-8850-s003.png]

# ***Tetramorium caespitum***

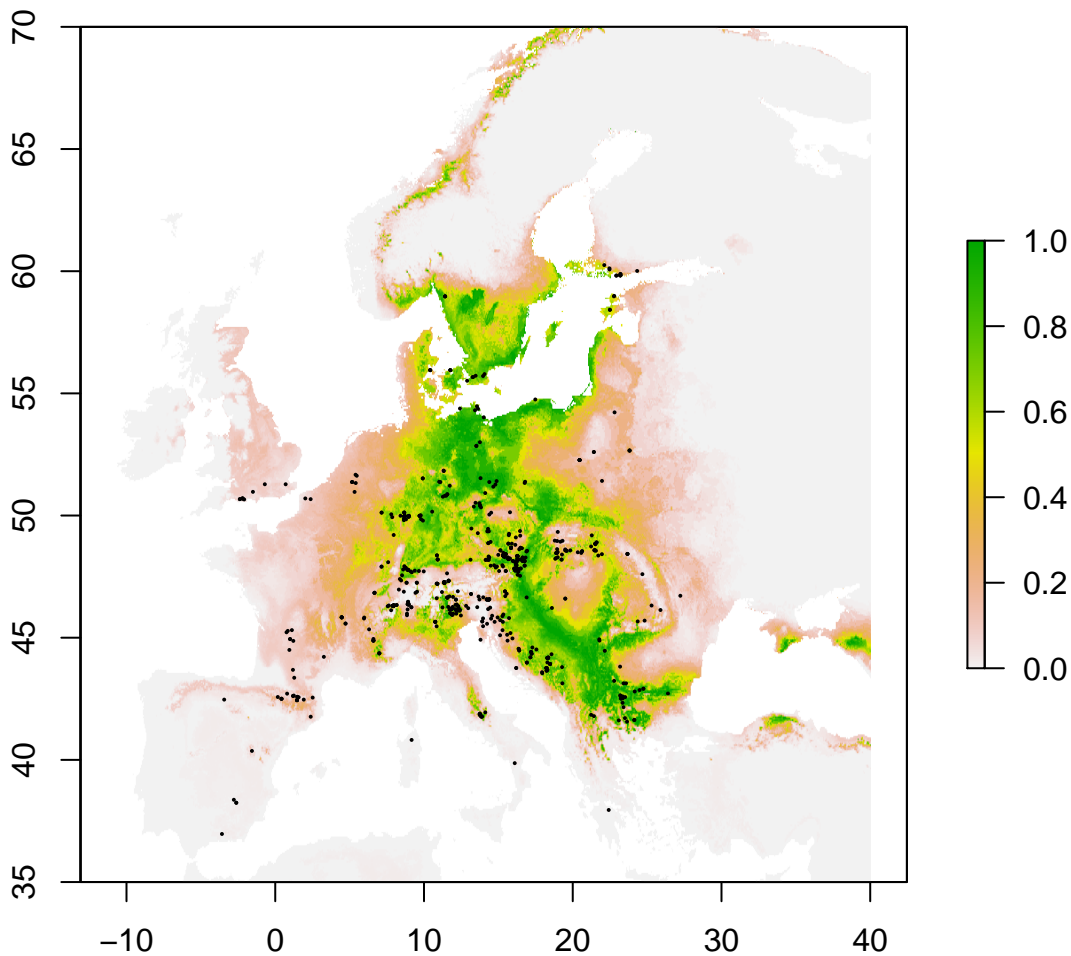

Supplement: Supplemental Information 8 — Illustrates that removing data from potentially questionable sources does not affect the conclusion of the model. [file peerj-08-8850-s008.pdf]
