# Supplementary material for: Monitoring the influx of new species through citizen science: the first introduced ant in Denmark
Source: PeerJ. 2020 Apr 8;8:e8850. doi: 10.7717/peerj.8850 (PMC7150537; doi:10.7717/peerj.8850)

**Supplemental material S2: Description of the Ant Hunt**

The Ant Hunt was a citizen science project that ran in Denmark from April 1^st^-October 1^st^ in 2017 and 2018, with a pilot testing in 2015. The project aimed to engage children age 8-11 in scientific discovery during school hours and especially outside school with their family. The project was run by the Natural History Museum of Denmark and the Center for Macroecology, Evolution and Climate at Copenhagen University. Participants were reached through touring the country, engaging families outside supermarkets and in collaboration with various museums and nature guides across the country. In order to participate, you had to buy an experimental kit, ensuring standardization across experiments. These kits cost 30 DKK during the first year and 50 DKK during the second.

The following protocol was included in the experimental kits used by all participants of the Ant Hunt. The original protocol can be found on the project website [www.myrejagten.dk](http://www.myrejagten.dk)

**Content**
This kit contains almost everything you need to carry out your Ant Hunt experiment. Besides the kit, all you need is five containers and five spoons from the kitchen to mix the liquid baits and some water, and then you are ready to begin. Enjoy.

**In the kit, you will find:**

• 8 g sugar (2 envelopes), to be dissolved in ½ dl water from the tap

• 1 g salt (1 envelope), to be dissolved in 1 dl water from the tap

• 10 g protein powder, to be dissolved in ½ dl water from the tap

• 10 ml extra virgin olive oil (1 envelope)

• 1 kammerjunker (a Danish cookie type)

• Cotton wool, to be divided into 5 pieces

• 6 labelled cardboard cards for the baits

• 7 wooden bbq sticks

• 1 nail

• 1 ’Experiment in progress’-sign

• 1 data sheet

• 6 ziploc bags

• 1 pair of tweezers

• 6 plastic vials (incl. 96 % alcohol) for sending in the ants

• 1 mini test-tube (without alcohol) for sending in extra ants, not caught on the baits

• Bubble wrap and paper napkins for secure packaging when sending in the ants

• 1 stamped cardboard envelope for sending in the ants

• 1 cardboard ant mask + elastic string

• 3 ant drawings for colouring

• 2 ant tattoos, semi-permanent

• 1 ant identification key

## **Prepare the experiment**

1. Mix the sugar water, saltwater and protein water in individual containers.
2. Divide the cotton wool into five balls and pierce each with a wooden bbq stick. Soak one cotton wool ball (on the stick) in saltwater, one in sugar water, one in protein water, one in oil and one in tap water.
3. Poke a hole in the middle of each bait card with the nail. Thereafter place the cotton wool balls with the sticks on their respectively labelled card, with the stick through the hole. The cotton wool balls must be soaked, but not dripping. It is important that the cotton wool is not squeezed.
4. Crumble the cookie (kammerjunker) into small crumbs and place them on the middle of the bait card with the text ‘Kammerjunker’ (cookie).

## **Carry out the experiment**

1. Choose a place where you have observed ants. Find a place where there is sunlight (also during the next couple of hours) and ideally, where there is a little cover from wind.
2. Place the ’Experiment in progress’-sign on the last wooden bbq stick and place it in the ground.
3. Carefully place the bait cards with the baits on the ground. Push the sticks a little into the ground, so that the cards do not blow away.
4. Note the time of the beginning of the experiment, locality and environmental data on the data sheet.
5. Give the ants time and space to find your baits. When the baits have been placed, they must be left for two hours. The baits are not allowed to be left out overnight. Check the baits regularly, to make sure the experiment is not destroyed and other animals do not eat the baits.

## **Collect the data and ants (two hours later)**

1. Count how many ants there are on each bait card, and write the number on the data sheet. If there are a lot of ants, estimate an approximate number. Do not disturb the ants, as they will run away. Also record if there are cards with no ants on them. Have a look around the area to see if there are any ants nearby. Catch a few and put them in the mini test tube. Note on the data sheet under ‘Comments – other observations’ where you found the extra ants. Freeze the ants in the mini tube, add 96 % alcohol and send them in with the rest of your catch.
2. Quickly collect the bait cards one at a time, picking up the cards with both the ants and the baits. Place each card in an individual ziploc bag. Close and secure each bag as soon as the ants and baits are in, but make sure there is a little air in the bag, so the ants are not squashed.
3. Put the bags with ants in the freezer overnight.

## **Report your results (the day after)**

1. Take out one bag of ants from the freezer at a time. For each bag, you have to: - Find the plastic vial with alcohol that matches the bait type in the bag. - Carefully sort out and discard the bait remnants and cotton wool. - Carefully count and record the number of ants on the data sheet. Use the tweezers to carefully place the ants in the plastic vial. Screw on the lid and make sure it is tight.
2. All plastic vials containing ants are placed in a ziploc bag with paper towels. The bag is then wrapped in bubble wrap, placed in the cardboard envelope and sent to the museum.
3. Type the data from the datasheet into the Ant Hunt database, found on [www.myrejagten.dk](http://www.myrejagten.dk). The scientists at the museum are looking forward to receiving and analysing your data and ants.


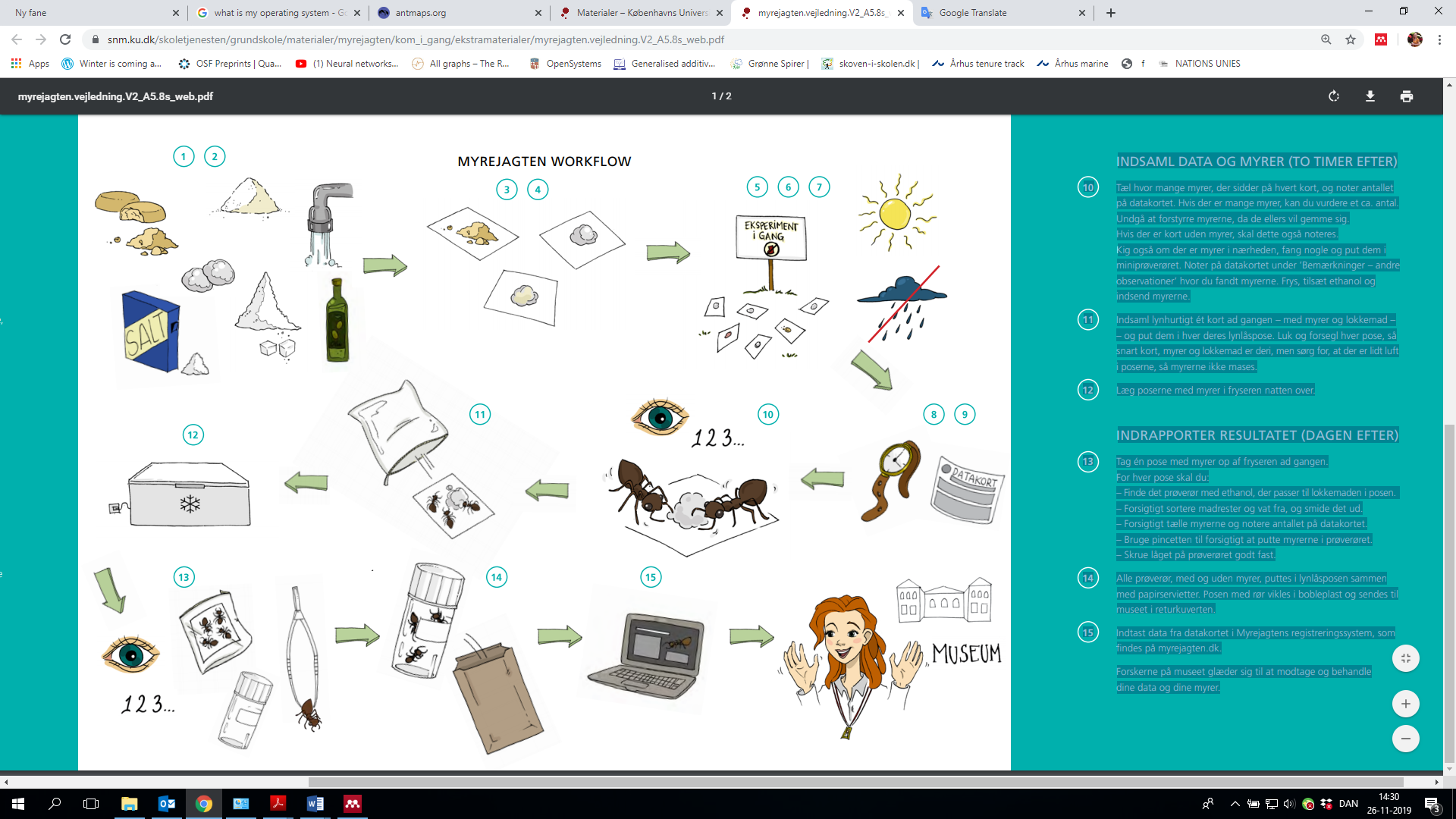

Supplement: Supplemental Information 4 [file peerj-08-8850-s004.docx]
